# Review of Recent Optofluidic Devices

**DOI:** 10.3390/mi17030291

**Published:** 2026-02-27

**Authors:** Aaron R. Hawkins

**Affiliations:** Electrical and Computer Engineering Department, Brigham Young University, Provo, UT 84602, USA; ahawkins@byu.edu

## 1. Introduction

The term “optofluidics” began appearing in scientific literature around 2002. This was followed by optofluidics-focused topical conferences and symposia, Special Issues in a variety of journals, and several books on the subject [1]. Optofluidics jelled at the intersection of microfluidics and integrated optics and tended toward devices and systems in miniaturized form, either on chip or in optical fiber. In order for something to be considered “optofluidic”, it needed fluid and light inputs and some type of interaction between the two. Since the field’s beginning, optofluidic development has tended to fall into three categories: (1) tunable photonic devices that use the changeable optical properties of a fluid as a means of varying an output; (2) labs-on-chip performing bioanalysis with light [2]; and (3) waveguides and optical fibers with a liquid component [3].

Almost 25 years after its origin, interest in optofluidics continues to be strong. As evidence, a Google Scholar search of papers published in 2025 with “optofluidic” in the title brings up nearly 3000 examples. Optofluidic research thrives in journals dedicated to optics, microfluidics, labs-on-chip, and miniaturization. Review articles on optofluidics now proliferate throughout scientific literature [4,5]. This particular review is meant to highlight recent optofluidics research published in *Micromachines*. As a journal focused on miniaturization and micro-scale devices, *Micromachines* is a natural fit for preserving and sharing optofluidics results. The papers highlighted in the next sections are divided into the same three major topics that have been used since the beginning to classify optofluidics research. Summaries are provided for each paper, including some of the illustrations that best explain the work. These papers are good examples of the flavor and type of innovation that flourishes in a field that shows no signs of slowing down.

## 2. Tunable Photonic Devices

**Figure 1:**
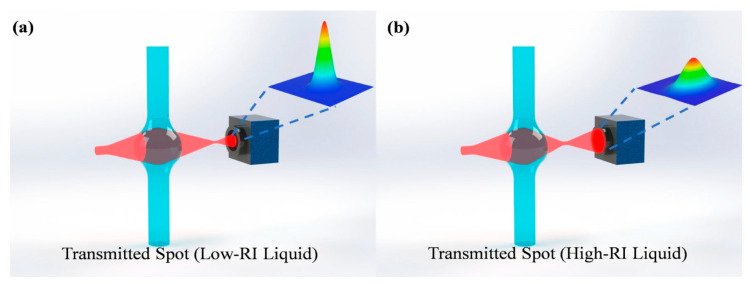
(**a**) A Gaussian beam passing through a low-refractive-index liquid. (**b**) A Gaussian beam passing through a high-refractive-index liquid. Figure taken from [6].

This section highlights research done in the area of tunable photonics. The first example describes a straightforward device that utilizes three elements: a fiber-coupled laser, a microfluidic channel consisting of a glass capillary tube attached to a glass base, and an imaging camera [6]. Along the glass tube, a “microbubble” is formed, which takes the shape of a lens. When fluids of different refractive indices are passed through the capillary, they effectively change the focal property of the lens. When a laser beam passes through this optical lens and is captured by a camera, the size and shape of the beam can be correlated to the refractive index of the fluid inside the capillary tube. The operating principle is illustrated in Figure 1. Listed advantages of this approach for measuring refractive index include low cost and small size, and these refractometers can potentially be deployed for environmental monitoring of water or analysis of a chemical stream. Authors report the detection of refractive index gradients as low as 1.4 × 10^−5^ within a refractive index range of 1.33 to 1.48.

**Figure 2:**
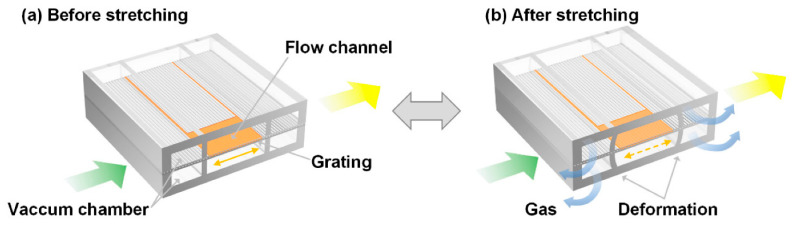
Schematic design of the optofluidic dye laser device and its tuning mechanism. The laser wavelength is adjusted before (**a**) and after (**b**) stretching the grating membrane using the vacuum force. Green: pump light; yellow: laser output. The orange line with dual arrows highlights the deformation of the grating film. Figure taken from [7].

Another example describes an optically pumped liquid dye laser built on a chip using polymethylsiloxane (PDMS) layers to form microchannels [7]. The PDMS layers include a grating layer with a 417 nm period made with nanoimprint lithography that can be stretched or compressed pneumatically to adjust the grating spacing. Tuning the grating in this way effectively tunes the distributed feedback cavity used to form a laser structure. The concept is illustrated in Figure 2. When tested with a dye consisting of 2 mM RhoG in DMSO and water, the effective index of the liquid channel remained higher (1.420) than the index of the surrounding PDMS layer (1.412) at the excitation wavelength. A 532 nm Nd:YAG pump laser operating with 5 ns pulses and a 1 Hz repetition rate produced emission around 589 nm. When the grating was tuned by pneumatic stretching, the wavelength varied by 7.84 nm with a resolution of 0.25 nm. The authors report a low threshold for this device of 164 nJ/pulse. This type of optically pumped dye laser is small and tunable, which makes it potentially valuable for bioanalysis applications.

## 3. Labs-on-Chip

**Figure 3:**
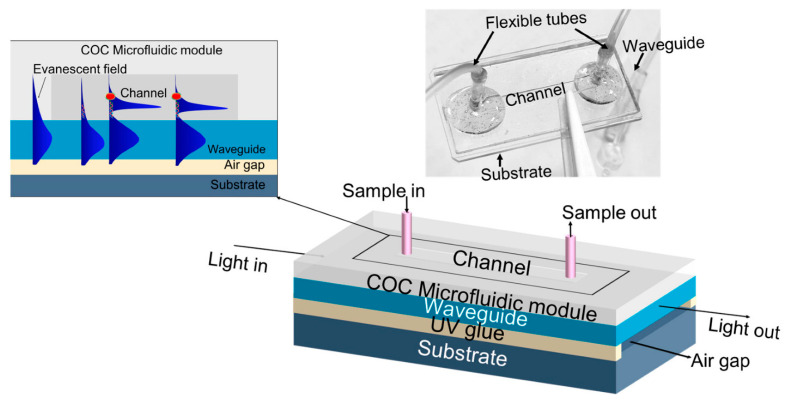
Waveguide biosensor: Schematic of the fabricated WG biosensor. Inset: Sensing mechanism and optical image of the fabricated device. Figure taken from [8].

This section highlights research involving bioanalysis applications. First, a lab-on-chip device made on glass substrates for the detection of DNA [8]. Fabrication is done through the bonding and gluing of layers. The most critical of these layers is a waveguide layer 0.2 mm thick. An air gap sits below the waveguide, and above is a liquid-filled channel, as illustrated in Figure 3. Liquid is injected into the channel through permanently attached tubes. The operating principle of this sensor depends on transmitting light through the waveguide layer. A small amount of light will interact with the liquid-filled channel through evanescence. The key to detecting DNA is attaching gold nanoparticles to the surface of the waveguide. These nanoparticles can collect specific DNA targets through bioconjugation. As more target DNA is captured, the intensity of transmitted light through the waveguide decreases due to plasmon resonance. The change in light transmission can be correlated to DNA concentration. Using a green LED as a light source, the authors report limits of detection of 33.1 fg/mL (4.36 fM) and a response time of 8 min.

Additionally, a device that is reminiscent of a lab-on-chip is constructed by sandwiching laser-cut double-sided tape between two PMMA layers [9]. Fluid channels 1 mm long and 10 mm wide were attached to electrodes and external fluid sources. Parallel channels were illuminated with a wide beam of 532 nm laser light, and the throughput from the channels was allowed to interfere. A resulting interference image made along the entire channel length is able to detect changes in refractive index in either of the channels. When fluids are passed through the channels, changes in chemical composition between a test and reference channel can be detected using the interference image. The idea of an optofluidic Young Interferometer was introduced earlier by the authors, and this latest paper reports on the combination of electrokinetic fluid transport with interference measurement. The authors used streptavidin as a test biomolecule and filled the fluid channels with an agarose gel. Tests were conducted with and without bovine serum albumin as an interferent.

A third bioanalysis example describes combining an optical fiber with microfluidic channels [10]. The channels are made from cast PDMS bonded to an ITO substrate, and the fiber is embedded within the PDMS walls during fabrication. Cells or microscale particles flow through a main channel and are deflected into sorting channels by the radiation pressure of light from the fiber. Cells or particles of different sizes and compositions will deflect different distances, allowing for continuous sorting. The laser coupled into the fiber operated at 980 nm with a power of around 180 mW. The authors report sorting yeast cells (8–10 μm in diameter) and polystyrene microspheres (15–10 μm in diameter) with 86% accuracy. They also report that 90% of the yeast cells remained intact after sorting.

## 4. Fiber-Based Devices

**Figure 4:**
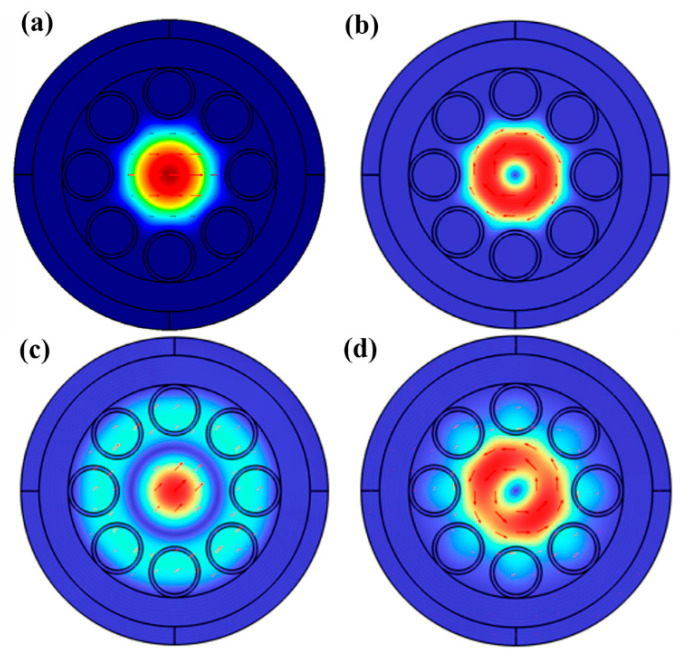
Modal intensity distributions of (**a**) HE11 and (**b**) TE01 core modes with ethanol only in the core and electric field distributions of (**c**) HE11 and (**d**) TE01 core modes with ethanol filled in the whole hollow area. Figure taken from [11].

This section highlights work focused on optofluidics applied to optical fibers. The first result is a theoretical exploration of a structured optical fiber filled with liquid in some or all of its hollow cross-sectional portions [11]. The paper specifically examines anti-resonant fibers and conditions in which the core alone is filled with liquid, along with conditions in which the core and surrounding hollow space are filled with liquid. Figure 4 illustrates some of the results from the models with optical modes highlighted for different use cases. Wall thicknesses are varied in the study, and the authors conclude that an optimal structure in terms of single-mode characteristics consists of 500 nm thick walls and a six-tube cladding. Losses are lower for fluid selectively confined to the fiber’s core.

A second highlighted work is another theoretical study of a fluid-filled optical fiber [12]. The cross-section of this fiber is composed of a fluid-filled core, a silicon layer of varying thickness, and a thick silica cladding. The motivation for studying this structure is a fiber-based analogy to slot waveguides formed on silicon substrates. The authors report transmission characteristics for different silicon thicknesses and core diameters. Under ideal conditions, a high fraction of power (56.3%) remains in the core. Raman gain parameters were also analyzed for the structures, and gains as high as 23.60 m^−1^W^−1^ were reported.

## 5. Outlook

In conclusion, this section explores some innovative research published outside of *Micromachines* that also points toward the future of optofluidics. One interesting development shows how optofluidics can fundamentally alter the fields from which it drew its inspiration. The authors go beyond traditional solid microfluidic channel confinement to use light to define microfluidic barriers through photothermal conversion [13]. This allows for dynamic, reconfigurable three-dimensional fluidic boundaries.

Another result demonstrates how optofluidics is being applied with new materials and platforms [14]. Researchers combine optofluidics concepts on low-cost paper templates. The optofluidic paper-based analytical device (PAD) consists of a macromolecule-driven flow (MDF) gate and photonic crystal (PhC) coding units. The intention is to mimic the function of an olfactory system.

Finally, two examples of the extensive reconfigurability capabilities of optofluidics are provided. The first demonstrates a microlens array (MLA) using fluid as the lens material [15]. By dynamically adjusting focal lengths, the optofluidic MLA provides a new strategy to break through the depth-of-field limitations for 3D imaging. A second noteworthy result uses all-optical fluidic components to form the basis of a bionic optical focusable imaging system [16]. The system exhibits a wide range of 2D field-of-view (FOV) tuning capability.
